# The Use of Fluorescent Anti-CEA Antibodies to Label, Resect and Treat Cancers: A Review

**DOI:** 10.3390/biom11121819

**Published:** 2021-12-02

**Authors:** Michael A. Turner, Thinzar M. Lwin, Siamak Amirfakhri, Hiroto Nishino, Robert M. Hoffman, Paul J. Yazaki, Michael Bouvet

**Affiliations:** 1 VA San Diego Healthcare System, La Jolla, CA 92161, USA; maturner@ucsd.edu (M.A.T.); siamirfakhri@health.ucsd.edu (S.A.); hnishino@ucsd.edu (H.N.); all@anticancer.com (R.M.H.); 2 Division of Surgical Oncology, Department of Surgery, University of California San Diego, La Jolla, CA 92037, USA; 3 Dana-Farber Cancer Institute, Boston, MA 02215, USA; thinzar_lwin@dfci.harvard.edu; 4 AntiCancer Inc., San Diego, CA 92111, USA; 5 Department of Immunology and Theranostics, Beckman Research Institute, City of Hope, Duarte, CA 91010, USA; pyazaki@coh.org

**Keywords:** carcinoembryonic antigen, CEA, fluorescence-guided surgery, FGS, infrared dyes, fluorescence, fluorescence labeling

## Abstract

A major barrier to the diagnosis and effective treatment of solid-tumor cancers is the difficulty in detection and visualization of tumor margins in primary and metastatic disease. The use of fluorescence can augment the surgeon’s ability to detect cancer and aid in its resection. Several cancer types express carcinoembryonic antigen (CEA) including colorectal, pancreatic and gastric cancer. Antibodies to CEA have been developed and tagged with near-infrared fluorescent dyes. This review article surveyed the use of CEA antibodies conjugated to fluorescent probes for in vivo studies since 1990. PubMed and Google Scholar databases were queried, and 900 titles and abstracts were screened. Fifty-nine entries were identified as possibly meeting inclusion/exclusion criteria and were reviewed in full. Forty articles were included in the review and their citations were screened for additional entries. A total of 44 articles were included in the final review. The use of fluorescent anti-CEA antibodies has been shown to improve detection and resection of tumors in both murine models and clinically. The cumulative results indicate that fluorescent-conjugated anti-CEA antibodies have important potential to improve cancer diagnosis and surgery. In an emerging technology, anti-CEA fluorescent antibodies have also been successfully used for photoimmunotherapy treatment for cancer.

## 1. Introduction

A major barrier to accurate cancer diagnosis and effective treatment is the inability to completely visualize the tumor. This is especially true of metastatic disease. Surgical resection remains a cornerstone of treatment for solid organ tumors. To achieve complete (R0) resection, the surgeon relies upon tactile and visual cues, preoperative imaging and his or her own experience [1,2]. The presence or absence of metastatic disease is critical in determining appropriate surgical treatment. The surgeon’s ability to accurately determine a tumor’s margin and identify metastatic lesions is paramount [3]. Failure to achieve R0 resection, often due to the invisibility of the tumor margin, is associated with disease recurrence [4,5]. Diagnostic laparoscopy is often used to detect metastatic disease, which can be essentially invisible, leading to unnecessary resection of the primary tumor. Cancer diagnosis and treatment can be greatly improved by making the “invisible” disease visible.

Fluorescence labeling of tumors can assist in achieving R0 resection and identifying metastatic lesions. Fluorescence is more sensitive than bright-light visualization and palpation in an intraoperative setting [3]. In fluorescence studies, the tumor-to-background-ratio (TBR) is an important concept. Fluorescence studies report the contrast ratio of the tumor signal and signal from normal tissue (background) to quantify results [1].

Initial studies focused on non-specific fluorescent dyes, relying on the enhanced permeability and retention (EPR) effect of tumors to create a contrast between tumor and normal tissue [2]. The most salient example of this is indocyanine green (ICG). ICG has been used for sentinel lymph node detection in breast [6] and gastric cancer [7], as well as liver tumor resection [8]. ICG is also starting to be used for head and neck surgery [9]. Oral administration of 5-aminolevulinic acid (5-ALA) is another fluorescent probe being used clinically. 5-ALA can help delineate malignant glioblastoma from normal brain tissue [10,11]. However, the lack of specificity of these probes limits their use in other types of cancer.

Recent studies use near-infrared (NIR) dyes, which improve tissue penetration due to lack of tissue autofluorescence. The NIR dyes are attached to a monoclonal antibody to a tumor antigen targeting the tumor of interest. One of the most common antibody targets is carcinoembryonic antigen (CEA).

CEA is a membrane-bound glycoprotein expressed in over 80% of colorectal cancers [12]. Although originally associated with colorectal cancer (CRC), it has subsequently been found in lung, breast, pancreatic, gallbladder, bladder, ovarian and gastric cancer [13]. CEA belongs to a family of glycoproteins called carcinoembryonic antigen cell adhesion molecules (CEACAM) and is also known as CEACAM5 and CD66e [14]. Several other CEACAMs have been validated as clinical biomarkers and therapeutic targets in addition to CEA [14].

In the present review, preclinical and clinical studies developing anti-CEA fluorescent antibodies for cancer diagnosis and treatment are reviewed. This review is broadly divided into CRC, pancreatic cancer and gastric cancer/other. Within each group, preclinical studies (subcutaneous, orthotopic and intraperitoneal (IP) murine models) and clinical studies using anti-CEA fluorescent antibodies are reviewed.

## 2. Materials and Methods

Two databases (PubMed and Google Scholar) were accessed in August 2021. Inclusion criteria included: (1) use of a specific anti-CEA probe with a fluorescent dye, (2) in vivo imaging, (3) original research study, (4) non-retracted and (5) accessible by the University of California, San Diego (UCSD) library. Exclusion criteria included: (1) nonspecific anti-CEA probes (probes to multiple targets), (2) articles that only reported ex vivo data, (3) review or opinion articles, (4) research articles using only nanobodies.

The phrases “fluorescence guided surgery AND carcinoembryonic antigen” and “fluorescence AND in vivo AND carcinoembryonic antigen” were used to query the PubMed database. The phrase “anti-Carcinoembryonic antigen AND fluorescence guided surgery AND in vivo” was used to query Google Scholar. All searches were limited to a publishing date on or after 1990 and “English” language.

**Figure 1:**
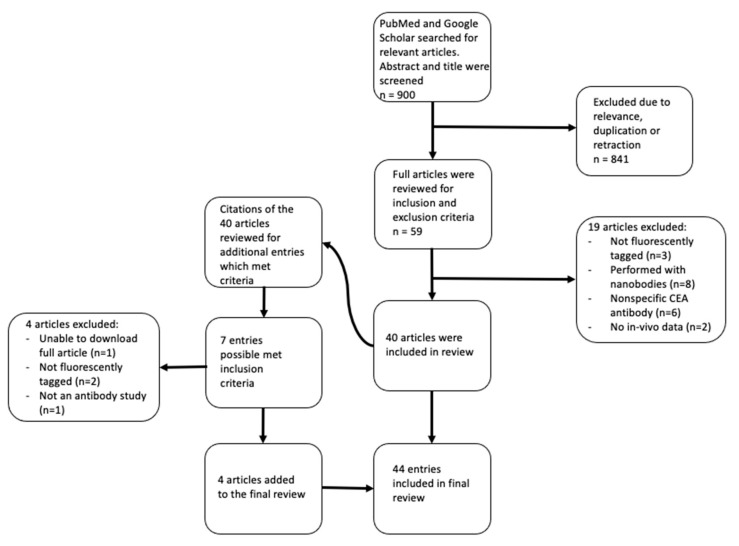
Search criteria and reasons for exclusion of articles.

A PubMed search resulted in 121 entries. A Google Scholar search resulted in 779 entries. Each abstract was screened for possible fit. After screening and removal of duplicates and retractions, 59 entries remained. These articles were read in full and 40 were included in the systematic review. The 40 articles then had their citations reviewed and an additional 8 entries were identified as possibly meeting inclusion criteria, of which 4 were included (Figure 1).

One author (MT) screened all the titles and abstracts, reviewed the citations, read all the articles that fit the inclusion criteria and decided which articles to include in which category. The search criteria and the articles selected were reviewed and approved by the several authors (T.M.L., S.A., H.N., P.J.Y, M.B.). Data extracted included: antibody name, dye name, dose of antibody-dye conjugate, time from injection to imaging/surgery, tumor type, animal model, number of subjects used, use of control and results of experiment including tumor-to-background ratio (TBR) (Table 1).

## 3. Results

### 3.1. Colorectal Cancer

#### 3.1.1. Subcutaneous Mouse Models

The first in vivo imaging study to evaluate an anti-CEA antibody conjugated to a fluorophore was performed by Pèlegrin et al. [15]. Human CRC cell line (T380) was used to establish subcutaneous tumors in mice which were then injected with MoAB 35 (CEA specific antibody) conjugated to fluorescein (non-specific IgG antibody was used as a control). Mice were imaged between 6 and 96 h, and at all time points, the fluorescence signal was greater in the tumor than liver or muscle. Pèlegrin et al. also used dye alone as a control. The authors found the tumor-to-liver ratio with MoAB 35-fluorescein was 120:1 compared to 1:1 with dye alone.

The most common human CRC cell line used to establish murine models was the CEA-positive, commercially available cell line, LS174T. In an LS174T subcutaneous murine model, Berk et al. used a fluorescently tagged anti-CEA antibody (ZCEO25) to quantify ligand-receptor density and to calculate an association constant confirming high affinity binding of the fluorescently labeled antibody to the tumor [16]. Lisy et al. [17] and Kaushal et al. [18] also investigated different anti-CEA fluorescent antibodies to LS174T subcutaneous murine models, showing higher TBR with anti-CEA fluorescent antibodies than either the control arm, nonspecific IgG conjugated dye, or a low CEA-expressing arm. Different CRC cell lines (e.g., SW1222, C15A3), with different anti-CEA fluorescent antibodies, showed similar successful results in subcutaneous murine models [19,20].

The subcutaneous models of CRC convincingly demonstrated that anti-CEA fluorescent antibodies can selectively label subcutaneous tumors and provided vital information regarding timing and dosing of different antibody-dye conjugates.

#### 3.1.2. Orthotopic and Intraperitoneal Mouse Models

Kaushal et al. reported the first use of anti-CEA florescent antibodies in a patient-derived orthotopic xenograft (PDOX) model of CRC (Colo4104) [18]. The PDOX tumor was brightly and specifically labeled by an anti-CEA fluorescent antibody compared to the nonspecific IgG control. Fluorescence-guided surgery (FGS) with anti-CEA fluorescent antibodies led to improved rates of R0 resection in a CRC PDOX model [21] and increased disease-free survival (DFS) and overall survival (OS) [22]. Anti-CEA fluorescent antibodies also labeled HT-29 cell line CRC tumors in orthotopic models with improved operative outcomes [23,24,25].

Other preclinical models can be created by tumor cell injection. Intraperitoneal (IP) murine CRC metastasis models established via IP injection and liver metastasis models established via CRC cell injection into the spleen (allowing them to “seed” the liver) have been used in anti-CEA fluorescent antibody studies [25]. Gutowski et al. [26] intraperitoneally injected mice with LS174T cells and demonstrated successful labeling of “very small” nodules (<1 mg in weight or <1 mm in diameter) with fluorescent anti-CEA antibody. Tumors as small as <3 mm were successfully resected. The authors reported a sensitivity of 90.7%, specificity of 97.2%, positive predictive value (PPV) of 94.7% and negative predictive value (NPV) of 94.9%. These results were confirmed in an LS174T intraperitoneal mouse model using a dual radio- and fluorescently labeled anti-CEA antibody [27]. Hekman et al. established a metastasis model created by injecting a human CRC cell line (GW-39) into the mouse lung [28]. The antibody-dye conjugate was able to detect early micrometastasis undetectable by bright light alone. FGS allowed for all fluorescent nodules to be resected with no fluorescence signal visualized in the post-resection tumor bed. Hiroshima et al. established CRC liver metastasis murine models via splenic HT-29 cell injection [25]. The anti-CEA fluorescent antibody allowed for detection of deep hepatic tumors. However liver background signal is an important limitation in liver fluorescence imaging [24]. Conjugating long polyethylene glycol chains to the dye (“PEGylation”), the antibody-dye had increased serum half-life and decreased liver signal [29]. Maawy et al. [29] demonstrated higher TBRs with lower hepatic signals in a PEGylated anti-CEA fluorescent antibody versus the non-PEGylated fluorescent antibody. There was also decreased signal in liver, lung, and lymph node using a PEGylated fluorescent dye conjugated to an anti-CEA antibody [29].

**Figure 2:**
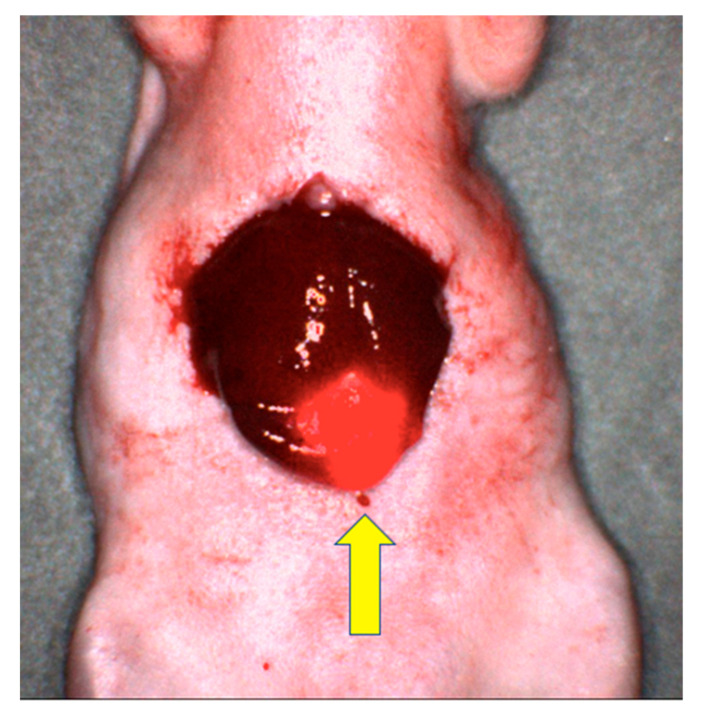
Image of LS174T liver metastasis in a mouse model after labeling with SGM-101. The yellow arrow points to the brightly labeled tumor with surrounding normal liver tissue [31].

SGM-101 was created from an anti-CEA antibody, SGM—ch511 conjugated to BM104, a fluorophore with an absorbance band centered at 700 nm [30]. Gutowski et al. [30] evaluated SGM-101 in 4 different murine models, 3 of which were CRC (LS174T intra-peritoneal, LS174T liver metastasis via spleen injection, HT29 cecal orthotopic). After resection of all tumors identified with single-photon emission computed tomography (SPECT) and visual inspection, the mice underwent FGS with NIR imaging, identifying and removing submillimeter tumor deposits. Our laboratory has established liver metastasis orthotopic models and shown that SGM-101 selectively labeled the tumor in the liver bed (Figure 2) [31]. SGM-101 is notable as the only anti-CEA fluorescent antibody in phase III clinical trials (NCT03659448 and NCT04642924).

Photoimmunotherapy (PIT) is a type of theranostics as it can both diagnose and treat disease. PIT utilizes a tumor-specific antibody conjugated to a photoactivatable dye to selectively bind cancer cells of interest and cause cell death when exposed to activating energy. Conventional photodynamic therapy (PDT) has limited use in cancer treatment due to lack of selective photosensitizers, limited tissue penetration and its reliance on reactive oxygen species (ROS) (problematic in solid tumors with a hypoxic milieu) [32,33]. However, recent studies using the NIR dye IR-700 conjugated to an anti-CEA antibody showed improved tissue penetration and cytotoxicity only when bound to a cell membrane, suggesting a different mechanism of action than ROS generation [32,33]. Elekonawo et al. used a CEA-expressing human CRC cell line, LoVo, subcutaneously implanted in 18 mice demonstrating labetuzumab-IR700 plus PIT significantly slowed tumor growth compared to PIT alone or labetuzumab-IR700 alone [34]. Hollandsworth et al. demonstrated similar findings in an LS174T orthotopic murine model [35].

#### 3.1.3. Clinical Trials

Clinical trials with fluorescent anti-CEA antibodies in CRC have been performed using SGM-101, as noted above. Initially, Boogerd et al. enrolled 26 patients with CRC into a safety and effectiveness study with SGM-101 [36]. Of the 9 patients in the dose escalation portion of the study, 4 of the 9 patients had an intraoperative signal (TBR = 1.83). An additional 3 patients had a fluorescence signal when the tumor was imaged after resection (the remaining 2 patients had complete pathological response). The other 17 patients had recurrent or peritoneal metastasis. After receiving SGM-101, a total of 44 malignant lesions were resected. Thirty-four of these lesions had an intraoperative fluorescence signal, and after excision, 43/44 lesions had a fluorescence signal. Importantly, 19 of these lesions were detected by fluorescence imaging only and were not clinically visible prior to NIR im-aging. Two false positives were recorded, one classified as dysplasia of the bladder urothelial lining and one as a peritoneal lesion containing blue ink particles due to endoscopic tattooing of the tumor. Neither of the false positives were CEA-positive by immunohistochemistry (IHC) staining. A total of 6 patients had their original treatment plan altered due to these findings in the clinical trial.

In 2021, de Valk et al. repeated the experiment with 37 patients and found similar optimal dose/timing and TBR with SGM-101 [37]. Seven true negative (no fluorescence, confirmed complete pathologic response after neoadjuvant therapy) and 2 false positives (one with CEA expressing mucin but no malignancy and one showing weak CEA ex-pression in the epithelial tissue) were recorded. Including primary and recurrent tumors and metastasis, a total of 97 lesions were resected, 49 of which were malignant. Of the 49 malignant lesions, 47 were fluorescent, although in the majority (27), the fluorescence signal was obscured due to anatomical positioning and only apparent after excision. Of the 48 benign lesions, 22 were false positives. Twelve patients had their original surgical plan altered due to fluorescence imaging, 9 of which were deemed appropriate (7 had additional tissue removed and 2 were downstaged due to lack of fluorescence signal and confirmed benign by frozen-section analysis). Schaap et al. conducted a non-randomized, multi-center, single-arm open-label study for patients with CRC peritoneal metastasis using SGM-101 [38]. Fourteen patients were scheduled to have a hyperthermic intraperitoneal chemotherapy (HIPEC) procedure and received SGM-101 prior to the procedure. Twelve patients had the HIPEC procedure (2 cases were aborted due to extensive, unresectable disease). The patients had their clinical peritoneal cancer index (PCI) calculated under bright light, then it was recalculated under fluorescence imaging (fPCI). Seven patients had their PCI increased due to fluorescence imaging, 4 of which were determined to be accurate based on histopathological analysis. In two patients, the PCI was incorrectly increased after fluorescence imaging (false positive fluorescent nodules confirmed to be benign by histopathological analysis). Histology of these false positives showed benign, hypervascularized, collagen-rich connective tissue with inflammatory changes. In one patient, the PCI decreased from 5 to 4 based on fluorescence imaging; however, histopathology showed that the PCI should have been 3 (1 false positive).

Folli et al. [39] used a different anti-CEA fluorescent antibody (CGP44290) in 6 patients with known CRC. After infusion with the anti-CEA fluorescent antibody, all 6 patients’ tumors became fluorescent. Keller et al. evaluated 27 patients with documented colonic polypoid lesions on a previous examination for a total of 33 colonic polypoid lesions (25 carcinomas, 8 adenomas) [40]. After an anti-CEA fluorescent antibody was applied directly to the lesions and allowed to incubate for 10 min, 19/25 carcinoma lesions and 3/8 adenomas were fluorescent. None of the surrounding normal tissue had a fluorescence signal. In reviewing the false negatives, bleeding or ulceration of the mucosa was a common finding and appeared to limit the sensitivity of fluorescent labeling. No false positives were recorded. Elekonawo et al. evaluated 10 patients, scheduled to undergo HIPEC for CRC peritoneal carcinomatosis and stratified them into dual radio- and fluorescence-labeled labetuzumab at 2 mg (n = 5) or 10 mg (n = 5) treatment arms [41]. Imaging of the resected lesions showed that 17/28 (61%) malignant lesions in the 2 mg group could be detected with fluorescence compared to 16/17 (95%) malignant lesions in the 10 mg group. However, the 10 mg group also had 4 false positives (3 of the lesions were classified as granulocytic inflammatory process with necrosis, fibrotic inflammation, and local colitis, while the 4th lesion was too damaged to undergo further histological analysis). No false positives were reported in the 2 mg group.

These clinical trials show the potential of fluorescent anti-CEA antibodies to augment intraoperative decision making. Ongoing phase III clinical trials may shed more light on which patients will most benefit from this emerging technology.

### 3.2. Pancreatic Cancer

#### 3.2.1. Subcutaneous Mouse Models

Kaushal et al. used an anti-CEA fluorescent monoclonal antibody to label 5 different human pancreatic cancer cell lines growing subcutaneously in nude mice, including BxPC3, a common pancreatic cancer cell line used in several FGS experiments [18]. All 5 subcutaneous tumor models had a specific fluorescence signal after receiving the antibody-dye conjugate. Animals with large tumors were selected to undergo tumor resection. After careful resection under a dissecting microscope with bright light, the tumor beds were imaged showing residual fluorescent disease in all mice (confirmed by histology). Knutson et al. also successfully labeled BxPC3 tumors in nude mice with an anti-CEA fluorescent antibody [42].

Maawy et al. compared PEGylated vs. non PEGylated anti-CEA fluorescent antibodies using a subcutaneous BxPC3 murine model [43]. The PEGylated dyes had a higher TBR than non-PEGylated dyes in both subcutaneous tumor models.

#### 3.2.2. Orthotopic and Intraperitoneal Mouse Models

Kaushal et al. used subcutaneous BxPC3 tumors to establish a pancreatic orthotopic murine model [18]. Small pancreatic tumors difficult to visualize under bright light, became obvious with fluorescence imaging after receiving fluorescent anti-CEA antibodies. Next, using IP pancreatic cancer cell injections to establish an intraperitoneal metastasis model, Kaushal et al. demonstrated peritoneal deposits, invisible by bright light, imaged brightly with fluorescence imaging after receiving fluorescent anti-CEA antibodies. In these experiments, a nonspecific IgG antibody-dye conjugate was used as a control and did not target the tumors. Similar studies confirmed the ability of anti-CEA fluorescent antibodies to selectively label BxPC3 orthotopic tumors [19,23,30,44,45]. Anti-CEA fluorescent antibodies in BxPC3 orthotopic models also have faster intraoperative tumor identification and improved sensitivity of intraperitoneal metastasis nodule identification with fluorescence imaging [46]. BxPC3 orthotopic models also demonstrated decreased local recurrence with improved DFS with FGS using anti-CEA fluorescent antibodies compared to bright-light surgery (BLS) alone [47]. FGS using anti-CEA fluorescent antibodies and neoadjuvant chemotherapy (NAC) also demonstrated improved R0 rates (92% vs. 45.5%), cure rates (40% vs. 4.5%), survival at 1 year (28% vs. 0%), median DFS (11 weeks vs. 5 weeks), and median OS (22 weeks vs. 13.5 weeks) in BxPC3 orthotopic models compared to BLS with NAC [48].

**Figure 3:**
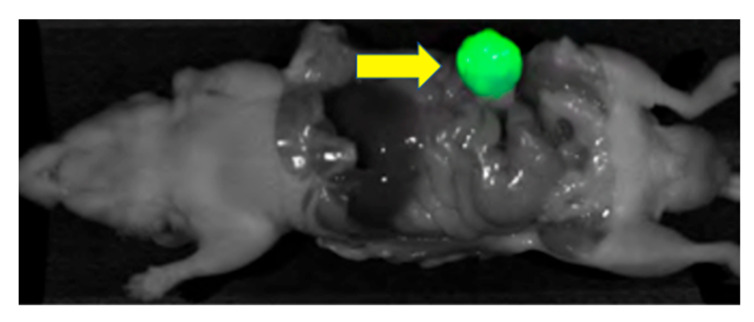
BxPC3 pancreatic orthotopic mouse model after receiving M5A-SW-IR800 (75 μg). The arrow points to the brightly labeled tumor [50].

Lwin et al. developed a humanized anti-CEA hT84.66-M5A-IR800m (M5A-IR800) fluorescence antibody to image green fluorescence protein (GFP) labeled BxPC3 pancreatic cancer orthotopic murine models [49]. M5A-IR800 had a stronger fluorescence signal than GFP labeling. Serial imaging between 6 and 72 h showed peak signal strength with M5A-IR800 in the orthotopic model at 48 h. As in the CRC experiments [24], there was high liver signal with M5A-IR800. To address this problem, Yazaki et al. conjugated the M5A antibody with long linear PEG molecules allowing 6–7 IR-800 dyes per antibody [50]. The new PEGylated M5A-sidewinder-IR800 (M5A-SW-IR800) had decreased hepatic accumulation and a longer serum half-life, resulting in decreased liver signal and an increased TBR in a BxPC3 pancreatic cancer orthotopic murine model (Figure 3).

Patient pancreatic tumors have also been used to establish orthotopic models. Using a patient tumor, Hiroshima et al. [51] investigated the efficacy of FGS with NAC in a CEA-negative, CA 19-9-positive pancreatic PDOX murine model. While the anti-CA 19-9 antibody-dye conjugate brightly labeled the PDOX tumor, the signal from the fluorescent anti-CEA antibody was “very weak”. In contrast, when Hiroshima et al. [52] used FGS with NAC in a CEA-positive pancreatic PDOX model, the tumors were labeled brightly. One in 8 mice in the FGS arm and 0/8 mice in the FGS + NAC had tumor recurrence 12 weeks after FGS compared to 6/8 in both the BLS and BLS + NAC arm. Lwin et al. used M5A-IR800 to image a pancreatic PDOX murine model using a patient’s liver metastasis from a pancreatic primary tumor and establishing it in the pancreas of a mouse [53]. M5A-IR800 brightly labeled the primary pancreatic tumor as well as the splenic and abdominal wall metastasis. Florescence signal was noted in the liver and bladder, with the average liver fluorescence signal 52% as strong as the average tumor signal.

In another example of theranostics, Maawy et al. used a chimeric anti-CEA antibody conjugated with IRDye 700DX NHS Ester to perform PIT in mice with BxPC3 orthotopic pancreatic cancer [54]. Mice received PIT and then were imaged weekly to assess tumor size. At the end of 5 weeks, the mice were euthanized, and tumor weight was recorded. Compared to control (PIT only), PIT with anti-CEA antibodies conjugated to IR700CW had significantly lower tumor weight at all time points. There was no difference in the weight of the mice minus the tumor between the two arms suggesting PIT was well tolerated.

#### 3.2.3. Clinical Trials

There is only one clinical trial with anti-CEA fluorescent antibodies for pancreatic cancer. Hoogstins et al. enrolled 12 patients with pancreatic ductal adenocarcinoma into the SGM-101 trial [55]. SGM-101 brightly labeled primary and metastatic tumors in 11 patients (one patient’s procedure was abandoned before visualizing the primary tumor due to the extent of metastatic disease). Seven of the primary tumors were resected (TBR 1.67 ± 0.37), 6 of which were confirmed adenocarcinoma (1 tumor sample was diagnosed as IPMN, a premalignant lesion, and considered a false positive). Three of the patients had peritoneal or liver metastasis. A total of 5 fluorescent, clinically suspicious nodules were removed, all demonstrated to be malignant with moderate-to-strong CEA expression (TBR 1.7 ± 0.42). An additional 8 non-fluorescent, clinically suspicious nodules were removed, 2 of which were malignant and thus classified as false negatives.

### 3.3. Gastric and Other Cancers

#### 3.3.1. Subcutaneous Mouse Models

Koga et al. [56] established HT1080 (human fibrosarcoma) and MKN45 (human gastric cancer cell line) subcutaneous murine models. The mice were injected with an anti-CEA fluorescent antibody which labeled both subcutaneous tumor models, albeit with high background signal. When subcellular imaging was performed on the resected tumor, the antibody-dye conjugate was mostly found on the surface of the cancer cells.

#### 3.3.2. Orthotopic and Intraperitoneal Mouse Models

Ito et al. [57] used a fluorescent anti-CEA antibody to compare the sensitivity of fluorescent imaging to MRI for the detection of peritoneal metastasis in gastric cancer cell lines. An anti-CEA antibody conjugated to ICG and NIR probe, XenoLight CF750, enabled detection of peritoneal tumor deposits in all four gastric cancer cell lines. The strength of the signal correlated to the level of CEA expression in the cell lines. While larger deposits (7 mm) could be detected by both fluorescent imaging and MRI, micrometastases < 2 mm were visualized only by fluorescence imaging, indicating that fluorescence is more sensitive than MRI for detecting small tumor deposits.

Shirasu et al. used an anti-CEA antibody conjugated with IRDye 700DX NHS Ester (termed 45IR) to study PIT with MKN-45-luc (luciferase-expressing human gastric adenocarcinoma) tumors [33]. Subcutaneous murine models were established and randomized into 4 groups: PIT + 45IR 200 µg, PIT + 45IR 100 µg, PIT + antibody without dye and PIT alone. One side of the mouse received PIT with the other side serving as internal control. The groups receiving PIT with 45IR had decreased fluorescence signal and tumor size (200 µg > 100 µg) compared to PIT + antibody without dye or PIT alone.

No clinical trials with fluorescent anti-CEA antibodies for gastric cancer were identified in this review (Table 1).

## 4. Discussion

One of the most commonly studied biomarkers for fluorescence labeling of tumors is CEA [58]. However, the majority of data are still in the preclinical stage. For CRC and pancreatic cancer, preclinical studies demonstrate fluorescent anti-CEA antibodies can selectively and brightly label tumors. Several FGS studies also demonstrate improved surgical outcomes (DFS and OS) in CRC and pancreatic cancer, though this still needs to be confirmed in clinical trials.

The lack of standard reporting of methods and results in the fluorescence studies complicates making direct comparisons [59]. However, in this review, the anti-CEA antibody-dye conjugates were analyzed for commonality in 3 components: antibody, dye and linker. The anti-CEA antibodies were either murine, chimeric or humanized monoclonal antibodies, but their CEA epitope specificity or affinity were not always disclosed. NIR fluorescent dyes (650–800 nm) are preferred based on their depth of penetration [44]. Development of new antibody-dye conjugates has been spurred by the commercial availability of the LICOR NHS-IR800CW dye and ease of amine chemistry conjugation. However, probe development is still in the early stage of development. Several groups have reported that the conjugated IR800 dye’s hydrophobicity can change the pharmacokinetics and biodistribution [50,60]. This has prompted using PEGylation of the antibody’s hinge domain to shield the IR800 dye’s hydrophobicity at the site of conjugation [50].

An important limitation of the preclinical data is that it is performed exclusively in immunocompromised mice. However, the CRC clinical trials and the sole clinical trial for pancreatic cancer confirm the main orthotopic murine model results. The clinical trials thus far have found no serious adverse effects in patients from the anti-CEA antibody-dye conjugate [36,37,39,55]. Future clinical trials are needed to confirm these findings. Currently, SGM-101 is the only anti-CEA antibody-dye conjugate in phase III trials (NCT03659448 and NCT04642924).

Another limitation is that the scope of this review only focuses on anti-CEA antibodies. There are several other targets showing great promise in fluorescence labeling including antibodies to TAG 72 [61], other CEACAMs [62], VEGF [63], and mucins, as well as several different delivery vehicles (e.g., nanobody [64], small particles [65]) and different labels (e.g., quantum dots [64]). Excellent reviews have already been done on these topics [1,2,58,59,66]. Given the absence of published head-to-head trials, no method is widely accepted as superior to the other. This review concludes that fluorescent anti-CEA antibodies improves tumor detection compared to bright light but does not speculate on its effectiveness compared to the other modalities used for FGS. Finally, only one author (M.A.T) screened the articles and decided which ones to include in the review. To modulate the risk of bias, the selection criteria and the articles chosen were reviewed and approved by several authors (T.M.L, S.A, H.N, P.J.Y, M.B).

Fluorescent anti-CEA antibodies are a promising diagnostic probe for CRC and pancreatic cancer. Further preclinical studies are necessary to determine its role in gastric cancer. Large clinical trials are necessary to further delineate its appropriate use in patients with CRC and pancreatic cancer.

## Figures and Tables

**Table 1:** List of anti-CEA fluorescent antibody studies.

Title	Author	Year	Model	Antibody	Dye	Dose	Timing	Tumor
Antibody-fluorescein conjugates for photoimmunodiagnosis of human colon carcinoma in nude mice [15]	Pèlegrin, A.	1991	Mice	MoAB 35 (Murine)	Fluorescein	20 μg	6–96 h	T380 (human CRC) subcutaneous (SQ)
Immunophotodiagnosis of colon carcinomas in patients injected with fluoresceinated chimeric antibodies against carcinoembryonic antigen [39]	Folli, S.	1992	Human	CGP44290 (Murine)	Fluorescein isothyocyanate	4.5 mg 9 mg	24 h	known primary CRC
Direct in vivo measurement of targeted binding in a human tumor xenograft [16]	Berk, D.A.	1997	Mice	ZCE025 (Murine)	Fluorescein	20–6000 μg	10 m–24 h	LS174T SQ
Intraoperative immunophotodetection for radical resection of cancers [26]	Gutowski, M.	2001	Mice	35A7 (Murine)	ICG	10, 40, 100 μg	48 h	LS174T IP cell injection
Fluorescence endoscopy using a fluorescein-labeled monoclonal antibody against carcinoembryonic antigen in patients with colorectal carcinoma and adenoma [40]	Keller, R.	2002	Human	Monoclonal (Murine)	FLOUS	2–6 mL	10 min	27 patients with large colonic lesions
In vivo near-infrared fluorescence imaging of carcinoembryonic antigen-expressing tumor cells in mice [17]	Lisy, M.	2008	Mice	Arcitumomab (Chimeric)	DY-676	40 μg	2–24 h	LS174T SQ
Fluorophore-conjugated anti-CEA antibody for the intraoperative imaging of pancreatic and colorectal cancer [18]	Kaushal, S.	2008	Mice	Monoclonal (Murine)	AlexaFluor 488	75 μg	24 h	BxPC3 SQ Colo4104 (CRC) SQ and cecal orthotopic
An LED light source and novel fluorophore combinations improve fluorescence laparoscopic detection of metastatic pancreatic cancer in orthotopic mouse models [45]	Metildi, C.	2012	Mice	Monoclonal (Murine)	Alexa 448 Alexa 555	75 μg	24 h	BxPC3 pancreatic orthotopic
Tumor-specific fluorescence antibody imaging enables accurate staging laparoscopy in an orthotopic model of pancreatic cancer [46]	Tran Cao, H.S.	2012	Mice	Monoclonal (Murine)	Alexa Fluor 488	75 μg	24 h	BxPC3 pancreatic orthotopic BxPC3 IP cell injection
New whole-body multimodality imaging of gastric cancer peritoneal metastasis combining fluorescence imaging with ICG-labeled antibody and MRI in mice [57]	Ito, A.	2013	Mice	HB 8747 (Murine)	XenoLight CF750	0.05 mg	10 m–7 d	MKN-28 (gastric cancer cell line), GCIY (gastric cancer cell line), GLM-1, GLM -2 (patient derived gastric cancer liver metastasis) IP models
Comparison of a chimeric anti-carcinoembryonic antigen antibody conjugated with visible or near-infrared fluorescent dyes for imaging pancreatic cancer in orthotopic nude mouse models [44]	Maawy, A.	2013	Mice	Monoclonal (Chimeric)	488 nm, 550 nm, 650 nm, 750 nm	50–75 μg (1.25 μmol of dye)	24 h	BxPC3 pancreatic orthotopic
SPECT- and fluorescence image-guided surgery using a dual-labeled carcinoembryonic antigen-targeting antibody [27]	Rijpkema, M.	2014	Mice	MN-14 (Murine)	IRDye 800CW	1–100 μg	1–8 d	LS174T SQ cell injection LS174T IP cell injection
Fluorescently labeled chimeric anti-CEA antibody improves detection and resection of human colon cancer in a patient-derived orthotopic xenograft (PDOX) nude mouse model [21]	Metildi, C.	2014	Mice	Monoclonal (Chimeric)	Alexa Fluor 488	75 μg	24 h	Patient derived (PD) CRC orthotopic
Successful fluorescence-guided surgery on human colon cancer patient-derived orthotopic xenograft mouse models using a fluorophore-conjugated anti-CEA antibody and a portable imaging system [22]	Hiroshima, Y.	2014	Mice	Monoclonal (Murine)	Alexa Fluor 488	Unspecified	24 h	PD CRC
Specific tumor labeling enhanced by polyethylene glycol linkage of near-infrared dyes conjugated to a chimeric anti-carcinoembryonic antigen antibody in a nude mouse model of human pancreatic cancer [43]	Maawy, A.	2014	Mice	Monoclonal (Chimeric)	DyLight 650/750 DyLight 650/750 PEG	2.5 nmol	5 m–24 h	BxPC3 SQ
Polyethylene glycol (PEG) linked to near-infrared (NIR) dyes conjugated to chimeric Anti-Carcinoembryonic Antigen (CEA) antibody enhances imaging of liver metastases in a nude-mouse model of human colon cancer [29]	Maawy, A.	2014	Mice	Monoclonal (Chimeric)	DyLight 650/750 DyLight 650/750 PEG	2.5 nmol (94 μg)	24 h	HT29 spleen injection
Advantages of fluorescence-guided laparoscopic surgery of pancreatic cancer labeled with fluorescent anti-carcinoembryonic antigen antibodies in an orthotopic mouse model [47]	Metildi, C.	2014	Mice	Monoclonal (Chimeric)	Alexa Fluor 488	75 μg	24 h	BxPC3-RFP pancreatic orthotopic
Fluorescence-guided surgery with a fluorophore-conjugated antibody to carcinoembryonic antigen (CEA), that highlights the tumor, improves surgical resection and increases survival in orthotopic mouse models of human pancreatic cancer [48]	Metildi, C.	2014	Mice	Monoclonal (Chimeric)	Alexa Fluor 488	75 μg	24 h	BxPC3-RFP pancreatic ortho
Potent and specific antitumor effect of CEA-targeted photoimmunotherapy [33]	Shirasu, N.	2014	Mice	C2-45 (Human)	IRDye 700DX NHS Ester	100, 200 μg	24 h	MKN-45-luc (gastric cancer) SQ cell injection
In vivo subcellular imaging of tumors in mouse models using a fluorophore-conjugated anti-carcinoembryonic antigen antibody in two-photon excitation microscopy [56]	Koga, S.	2014	Mice	CB30 (Murine)	Alexa 594	10, 50 μg	24 h	HT1080 (human fibrosarcoma) SQ cell injection MKN45 (human gastric) SQ cell injection
Metastatic recurrence in a pancreatic cancer patient-derived orthotopic xenograft (PDOX) nude mouse model is inhibited by neoadjuvant chemotherapy in combination with fluorescence-guided surgery with an anti-CA 19-9-conjugated fluorophore. [51]	Hiroshima, Y.	2014	Mice	Monoclonal (Chimeric)	DyLight 650	50 μg	24 h	PD pancreatic cancer patient-derived orthotopic xenograft (PDOX)
Fluorescence-guided surgery, but not bright-light surgery, prevents local recurrence in a pancreatic cancer patient-derived orthotopic xenograft (PDOX) model resistant to neoadjuvant chemotherapy (NAC) [52]	Hiroshima, Y.	2015	Mice	Monoclonal (Chimeric)	DyLight 650	50 μg	24 h	PD pancreatic cancer PDOX
Near-infrared photoimmunotherapy with anti-CEA-IR700 results in extensive tumor lysis and a significant decrease in tumor burden in orthotopic mouse models of pancreatic cancer [54]	Maawy, A.	2015	Mice	Monoclonal (Chimeric)	IRDye 700DX NHS Ester	100 μg	24 h	BxPC3 pancreatic orthotopic
Preclinical evaluation of a novel CEA-targeting near-infrared fluorescent tracer delineating colorectal and pancreatic tumors [23]	Boonstra, M.	2015	Mice	ssSM3E (Humanized)	IRDye 800CW	28 μg	8–120 h	HT29 cecal orthotopic BxPC3 cell injection pancreatic ortho
Effective fluorescence-guided surgery of liver metastasis using a fluorescent anti-CEA antibody [25]	Hiroshima, Y.	2016	Mice	Monoclonal (Chimeric)	DyLight 650	50 μg	24 h–72 h	HT29 liver orthotopic and liver metastasis spleen injection
Development and evaluation of a fluorescent antibody-drug conjugate for molecular imaging and targeted therapy of pancreatic cancer [42]	Knutson, S.	2016	Mice	Monoclonal (Murine)	DyLight-680-4xPEG	100 μg	24 h	BxPC3 SQ
Near-infrared-conjugated humanized anti-carcinoembryonic antigen antibody targets colon cancer in an orthotopic nude-mouse model [24]	DeLong, J.	2017	Mice	M5A (Humanized)	NHS-IRDye 800CW	75 μg	24–48 h	HT29 cecal orthotopic
Detection of micrometastases using SPECT/fluorescence dual-modality imaging in a CEA-expressing tumor model [28]	Hekman, M.	2017	Mice	Labetuzumab (Humanized)	IRDye 800CW	10 μg	3 d	GW-39 (human CRC) cell injection lung metastasis
SGM-101: An innovative near-infrared dye-antibody conjugate that targets CEA for fluorescence-guided surgery [30]	Gutowski, M.	2017	Mice	SGM-ch511 (Chimeric)	BM104	30 μg	24 h–96 h	LS174T IP model HT29 cecal orthotopic LS174T liver metastasis via spleen BxPC3 pancreatic orthotopic
A Dual Reporter Iodinated Labeling Reagent for Cancer Positron Emission Tomography Imaging and Fluorescence-Guided Surgery [20]	Lu, Z.	2018	Mice	A5B7 (Murine)	I-Green	44 μg	72 h	SW1222 CRC SQ
Safety and effectiveness of SGM-101, a fluorescent antibody targeting carcinoembryonic antigen, for intraoperative detection of colorectal cancer: a dose-escalation pilot study [36]	Boogerd, L.S.F.	2018	Human	SGM-ch511 (Chimeric)	BM104	5–10 mg	48 h–96 h	CRC
Image-guided surgery in patients with pancreatic cancer: First results of a clinical trial using SGM-101, a novel carcinoembryonic antigen-targeting, near-infrared fluorescent agent [55]	Hoogstins, C.	2018	Human	SGM-ch511 (Chimeric)	BM104	5, 7.5, 10 mg	48 h, 96 h	PDAC
Fluorescent humanized anti-CEA antibody specifically labels metastatic pancreatic cancer in a patient-derived orthotopic xenograft (PDOX) mouse model [53]	Lwin, T.M.	2018	Mice	M5A (Humanized)	IRDye 800CW	75 μg	48 h	PD pancreatic PDOX
Tumor-specific labeling of pancreatic cancer using a humanized anti-CEA antibody conjugated to a near-infrared fluorophore [49]	Lwin, T.M.	2018	Mice	M5A (Humanized)	IRDye 800CW	75 μg	6–96 h	BxPC3 pancreatic orthotopic
Improved antibody-guided surgery with a near-infrared dye on a pegylated linker for CEA-positive tumors [50]	Yazaki, P.J.	2019	Mice	M5A (Humanized)	NHS-IRDye 800CW PEGylated	75 μg	24–96 h	BxPC3 pancreatic orthotopic
Carcinoembryonic antigen-targeted photodynamic therapy in colorectal cancer models [34]	Elekonawo, F.	2019	Mice	Labetuzumab (Humanized)	IRDye 700DX NHS Ester	30 μg		LOVO SQ cell injection
Near-infrared photoimmunotherapy is effective treatment for colorectal cancer in orthotopic nude-mouse models [35]	Hollandsworth, H.M.	2020	athymic nude mice	M5A (Humanized)	IRDye 700DX NHS Ester	50 μg	24 h	LS174T cecal orthotopic
Carcinoembryonic antigen-specific, fluorescent image-guided cytoreductive surgery with hyperthermic intraperitoneal chemotherapy for metastatic colorectal cancer [38]	Schaap, D.P.	2020	Human	SGM-ch511 (Chimeric)	BM104	10–15 mg	4–6 d	peritoneal metastatic CRC
Multimodal image-guided surgery of colorectal peritoneal carcinomatosis: a phase 1 clinical trial [41]	Elekonawo, F.	2020	Human	Labetuzumab (Humanized)	IRDye 800CW	2 or 5 mg	5–6 days	Colorectal peritoneal metastasis
Near-infrared fluorescent imaging of pancreatic cancer in mice using a novel antibody to CEACAM 5 [19]	Zhou, X.	2021	Mice	C1P83	IRDye 800CW	25 μg (SQ model) 25, 100 μg (ortho)	24 h–6 d	C15A3 CRC SQ BxPC3 pancreatic orthotopic
Spectrally distinct double labeling of colon-cancer liver metastases and adjacent liver segment with a near-infrared-labeled anti-Carcinoembryonic antigen (CEA_ antibody and indocyanine green in an orthotopic mouse model. [31]	Nishino, H.	2021	Mice	SGM-ch511 (Chimeric)	BM104	Unspecified	48 h–96 h	LS174T liver orthotopic
Dose-finding study of a CEA-targeting agent, SGM-101, for intraoperative fluorescence imaging of colorectal cancer [37]	de Valk, K.	2021	Human	SGM-ch511 (Chimeric)	BM104	5–15 mg	24 h–6 d	CRC

## Data Availability

Not applicable.
